# 4-[5-(Pyridin-4-yl)-1,3,4-oxadiazol-2-yl]pyridinium benzoate

**DOI:** 10.1107/S1600536811001358

**Published:** 2011-01-22

**Authors:** Meng Ting Han, Yuan Zhang

**Affiliations:** a Ordered Matter Science Research Center, College of Chemistry and Chemical, Engineering, Southeast UniVersity, Nanjing 211189, People’s Republic of China

## Abstract

In the title compound, C_12_H_9_N_4_O^+^·C_7_H_5_O_2_^−^, π–π stacking inter­actions [centroid–centroid distance = 3.6275 (14)  Å] stabilize the crystal structure. The dihedral angles between the central ring and the terminal rings are 3.27 (12) and 10.30 (13)°.

## Related literature

For background to the development of ferroelectric compounds, see: Haertling *et al.* (1999 ▶); Homes *et al.* (2001 ▶). For the synthesis of a variety of compounds with potential piezoelectric and ferroelectric properties, see: Ye *et al.* (2006 ▶); Zhang *et al.* (2008 ▶). 
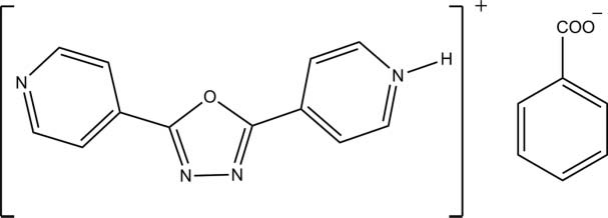


## Experimental

### 

#### Crystal data

C_12_H_9_N_4_O^+^·C_7_H_5_O_2_^−^
*M* *_r_* = 346.34
Monoclinic, 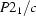

*a* = 20.459 (4) Å
*b* = 7.1958 (14) Å
*c* = 11.249 (2) Å
β = 90.53 (3)°
*V* = 1656.0 (5) Å^3^
*Z* = 4
Mo *K*α radiation
μ = 0.10 mm^−1^
*T* = 293 K
0.20 × 0.20 × 0.20 mm

#### Data collection

Rigaku Mercury2 diffractometer
Absorption correction: multi-scan (*CrystalClear*; Rigaku, 2005 ▶) *T*_min_ = 0.854, *T*_max_ = 1.000
16725 measured reflections
3808 independent reflections
2248 reflections with *I* > 2σ(*I*)
*R*_int_ = 0.064

#### Refinement

*R*[*F* ^2^ > 2σ(*F* ^2^)] = 0.059
*wR*(*F* ^2^) = 0.171
*S* = 1.02
3808 reflections
236 parameters
H-atom parameters constrained
Δρ_max_ = 0.27 e Å^−3^
Δρ_min_ = −0.28 e Å^−3^

### 

Data collection: *CrystalClear* (Rigaku, 2005 ▶); cell refinement: *CrystalClear*; data reduction: *CrystalClear*; program(s) used to solve structure: *SHELXS97* (Sheldrick, 2008 ▶); program(s) used to refine structure: *SHELXL97* (Sheldrick, 2008 ▶); molecular graphics: *SHELXTL* (Sheldrick, 2008 ▶); software used to prepare material for publication: *SHELXTL*.

## Supplementary Material

Crystal structure: contains datablocks I, global. DOI: 10.1107/S1600536811001358/jh2244sup1.cif

Structure factors: contains datablocks I. DOI: 10.1107/S1600536811001358/jh2244Isup2.hkl

Additional supplementary materials:  crystallographic information; 3D view; checkCIF report

## Figures and Tables

**Table 1:** Hydrogen-bond geometry (Å, °)

*D*—H⋯*A*	*D*—H	H⋯*A*	*D*⋯*A*	*D*—H⋯*A*
N1—H1*A*⋯O2^i^	0.86	1.79	2.648 (2)	174
C8—H8⋯O1^ii^	0.93	2.48	3.371 (3)	161

Symmetry codes: (i) 
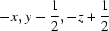
; (ii) 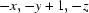.

**Fig. 1.**
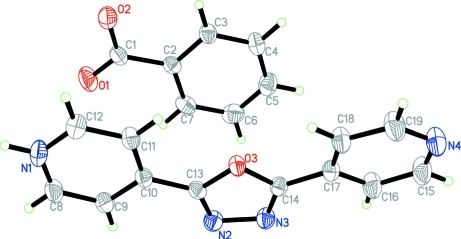
Perspective structure of the title compound, showing the atom-numbering scheme. Displacement ellipsoids are drawn at the 30% probability level.

## supplementary crystallographic information

### Comment

At present, much attention in ferroelectric material field is focused on developing ferroelectric pure organic or inorganic compounds (Haertling *et al.* 1999; Homes *et al.* 2001). Recently we have reported the synthesis of a variety of compounds (Ye *et al.*, 2006; Zhang *et al.*, 2008), which have potential piezoelectric and ferroelectric properties. In order to find more dielectric ferroelectric materials, we investigate the physical properties of the title compound(Fig. 1). The dielectric constant of the title compound as a function of temperature indicates that the permittivity is basically temperature-independent (dielectric constant equaling to 3.6 to 4.7), suggesting that this compound should be not a real ferroelectrics or there may be no distinct phase transition occurred within the measured temperature range. Similarly, below the melting point (374 K) of the compound, the dielectric constant as a function of temperature also goes smoothly, and there is no dielectric anomaly observed (dielectric constant equaling to 3.0 to 4.2).Herein, we report the synthesis and crystal structure of the title compound.

The asymmetric unit of (I) consists of one bpo cation and one benzoate anions. The π—π packing interaction of adjacent rings with *Cg*(1)—*Cg*(4), 3.6275 (14) A °; *Cg*(3)—*Cg*(3), 4.1148 (16) A °; [*Cg*(1), *Cg*(3) and *Cg*(4) are the centroids of rings, where *Cg*(1): O3/C13/N2/N3/C14; *Cg*(3): N4/C15–C19; *Cg*(4): C2–C7;],make great contribution to the stability of the crystal structure.

### Experimental

A mix of 2,5-bis(4-pyridyl)-1,3,4-oxadiazole (2.24 g, 0.01 mol) and benzoate acid (2.44 g,0.02 mol) in methanol (20 ml) was stirred until clear. After several days, the title compound was formed and recrystallized from solution to afford colourlesss prismatic crystals suitable for X-ray analysis.

### Refinement

H atoms were positioned geometrically and refined using a riding model, with C—H = 0.93 Å and *U*_iso_(H) = 1.2_eq_(C).

### Crystal data

**Table:**

C_12_H_9_N_4_O^+^·C_7_H_5_O_2_^−^	*F*(000) = 720
*M**_r_* = 346.34	*D*_x_ = 1.389 Mg m^−^^3^
Monoclinic, *P*2_1_/*c*	Mo *K*α radiation, λ = 0.71073 Å
Hall symbol: -P 2ybc	Cell parameters from 3808 reflections
*a* = 20.459 (4) Å	θ = 2.6–27.5°
*b* = 7.1958 (14) Å	µ = 0.10 mm^−^^1^
*c* = 11.249 (2) Å	*T* = 293 K
β = 90.53 (3)°	Prism, colourless
*V* = 1656.0 (5) Å^3^	0.20 × 0.20 × 0.20 mm
*Z* = 4	

### Data collection

**Table:**

Rigaku Mercury2 diffractometer	3808 independent reflections
Radiation source: fine-focus sealed tube	2248 reflections with *I* > 2σ(*I*)
graphite	*R*_int_ = 0.064
Detector resolution: 13.6612 pixels mm^-1^	θ_max_ = 27.5°, θ_min_ = 3.0°
CCD_Profile_fitting scans	*h* = −26→26
Absorption correction: multi-scan (*CrystalClear*; Rigaku, 2005)	*k* = −9→9
*T*_min_ = 0.854, *T*_max_ = 1.000	*l* = −14→14
16725 measured reflections	

### Refinement

**Table:**

Refinement on *F*^2^	Secondary atom site location: difference Fourier map
Least-squares matrix: full	Hydrogen site location: inferred from neighbouring sites
*R*[*F*^2^ > 2σ(*F*^2^)] = 0.059	H-atom parameters constrained
*wR*(*F*^2^) = 0.171	*w* = 1/[σ^2^(*F*_o_^2^) + (0.079*P*)^2^ + 0.2253*P*] where *P* = (*F*_o_^2^ + 2*F*_c_^2^)/3
*S* = 1.02	(Δ/σ)_max_ < 0.001
3808 reflections	Δρ_max_ = 0.27 e Å^−^^3^
236 parameters	Δρ_min_ = −0.28 e Å^−^^3^
0 restraints	Extinction correction: *SHELXL97* (Sheldrick, 2008), Fc^*^=kFc[1+0.001xFc^2^λ^3^/sin(2θ)]^-1/4^
Primary atom site location: structure-invariant direct methods	Extinction coefficient: 0.0130 (18)

### Special details

**Table:**

Geometry. All e.s.d.'s (except the e.s.d. in the dihedral angle between two l.s. planes) are estimated using the full covariance matrix. The cell e.s.d.'s are taken into account individually in the estimation of e.s.d.'s in distances, angles and torsion angles; correlations between e.s.d.'s in cell parameters are only used when they are defined by crystal symmetry. An approximate (isotropic) treatment of cell e.s.d.'s is used for estimating e.s.d.'s involving l.s. planes.
Refinement. Refinement of *F*^2^ against ALL reflections. The weighted *R*-factor *wR* and goodness of fit *S* are based on *F*^2^, conventional *R*-factors *R* are based on *F*, with *F* set to zero for negative *F*^2^. The threshold expression of *F*^2^ > σ(*F*^2^) is used only for calculating *R*-factors(gt) *etc*. and is not relevant to the choice of reflections for refinement. *R*-factors based on *F*^2^ are statistically about twice as large as those based on *F*, and *R*- factors based on ALL data will be even larger.

### Fractional atomic coordinates and isotropic or equivalent isotropic displacement parameters (Å^2^)

**Table:**

	*x*	*y*	*z*	*U*_iso_*/*U*_eq_	
C1	0.11341 (11)	0.8827 (3)	0.1577 (2)	0.0468 (5)	
C2	0.18546 (10)	0.8838 (3)	0.13768 (18)	0.0406 (5)	
C3	0.22890 (10)	0.9438 (3)	0.22500 (19)	0.0453 (5)	
H3	0.2133	0.9853	0.2977	0.054*	
C4	0.29500 (11)	0.9420 (3)	0.2041 (2)	0.0529 (6)	
H4	0.3240	0.9831	0.2626	0.064*	
C5	0.31834 (12)	0.8796 (3)	0.0974 (2)	0.0602 (7)	
H5	0.3631	0.8780	0.0839	0.072*	
C6	0.27599 (13)	0.8197 (3)	0.0105 (2)	0.0625 (7)	
H6	0.2921	0.7771	−0.0615	0.075*	
C7	0.20926 (12)	0.8224 (3)	0.02954 (19)	0.0510 (6)	
H7	0.1805	0.7831	−0.0299	0.061*	
C8	0.04872 (11)	0.3443 (3)	0.0797 (2)	0.0566 (6)	
H8	0.0171	0.3137	0.0231	0.068*	
C9	0.11320 (10)	0.3396 (3)	0.0482 (2)	0.0494 (6)	
H9	0.1251	0.3092	−0.0290	0.059*	
C10	0.16028 (10)	0.3805 (3)	0.13262 (18)	0.0398 (5)	
C11	0.14062 (10)	0.4269 (3)	0.2463 (2)	0.0471 (6)	
H11	0.1712	0.4538	0.3054	0.057*	
C12	0.07469 (11)	0.4324 (3)	0.2698 (2)	0.0545 (6)	
H12	0.0612	0.4662	0.3455	0.065*	
C13	0.22928 (10)	0.3753 (3)	0.10114 (18)	0.0391 (5)	
C14	0.33195 (10)	0.3951 (3)	0.12671 (18)	0.0401 (5)	
C15	0.50844 (12)	0.4366 (4)	0.2044 (3)	0.0739 (8)	
H15	0.5481	0.4410	0.1649	0.089*	
C16	0.45215 (11)	0.4228 (4)	0.1364 (2)	0.0634 (7)	
H16	0.4541	0.4184	0.0539	0.076*	
C17	0.39284 (10)	0.4158 (3)	0.19378 (19)	0.0430 (5)	
C18	0.39322 (11)	0.4232 (3)	0.3154 (2)	0.0572 (7)	
H18	0.3542	0.4195	0.3572	0.069*	
C19	0.45201 (13)	0.4362 (4)	0.3748 (2)	0.0721 (8)	
H19	0.4513	0.4400	0.4575	0.086*	
N1	0.02979 (9)	0.3912 (3)	0.1885 (2)	0.0577 (6)	
H1A	−0.0111	0.3947	0.2058	0.069*	
N2	0.25515 (9)	0.3426 (3)	−0.00105 (16)	0.0489 (5)	
N3	0.32318 (9)	0.3568 (3)	0.01599 (16)	0.0493 (5)	
N4	0.50991 (10)	0.4439 (3)	0.3221 (2)	0.0726 (7)	
O1	0.07395 (8)	0.8483 (3)	0.08005 (16)	0.0711 (5)	
O2	0.09672 (7)	0.9211 (2)	0.26772 (15)	0.0629 (5)	
O3	0.27482 (6)	0.40949 (19)	0.18688 (12)	0.0406 (4)	

### Atomic displacement parameters (Å^2^)

**Table:**

	*U*^11^	*U*^22^	*U*^33^	*U*^12^	*U*^13^	*U*^23^
C1	0.0439 (13)	0.0411 (12)	0.0553 (15)	−0.0043 (10)	−0.0113 (11)	−0.0002 (10)
C2	0.0444 (12)	0.0344 (11)	0.0429 (12)	−0.0029 (9)	−0.0044 (9)	0.0034 (9)
C3	0.0413 (12)	0.0522 (13)	0.0422 (12)	−0.0002 (10)	−0.0042 (9)	0.0024 (10)
C4	0.0401 (13)	0.0595 (15)	0.0591 (15)	−0.0068 (11)	−0.0098 (11)	0.0076 (11)
C5	0.0448 (14)	0.0601 (16)	0.0758 (18)	−0.0034 (12)	0.0100 (13)	0.0098 (13)
C6	0.0682 (18)	0.0578 (16)	0.0618 (16)	−0.0058 (13)	0.0178 (13)	−0.0026 (12)
C7	0.0615 (15)	0.0460 (13)	0.0456 (13)	−0.0091 (11)	−0.0039 (11)	0.0015 (10)
C8	0.0395 (13)	0.0617 (15)	0.0682 (17)	−0.0021 (11)	−0.0132 (12)	−0.0008 (12)
C9	0.0428 (13)	0.0561 (14)	0.0492 (13)	0.0003 (10)	−0.0092 (10)	−0.0042 (11)
C10	0.0370 (11)	0.0373 (11)	0.0451 (12)	−0.0022 (9)	−0.0050 (9)	0.0046 (9)
C11	0.0388 (12)	0.0560 (14)	0.0464 (13)	−0.0034 (10)	−0.0063 (10)	0.0027 (10)
C12	0.0451 (13)	0.0617 (16)	0.0569 (15)	0.0032 (11)	0.0027 (11)	0.0064 (11)
C13	0.0407 (11)	0.0394 (12)	0.0372 (11)	−0.0030 (9)	−0.0074 (9)	−0.0002 (9)
C14	0.0353 (11)	0.0432 (12)	0.0419 (12)	−0.0020 (9)	0.0049 (9)	0.0026 (9)
C15	0.0372 (14)	0.103 (2)	0.081 (2)	−0.0078 (14)	0.0073 (13)	−0.0025 (16)
C16	0.0410 (13)	0.092 (2)	0.0574 (15)	−0.0070 (13)	0.0037 (11)	0.0018 (13)
C17	0.0362 (11)	0.0467 (13)	0.0460 (13)	−0.0023 (9)	0.0008 (9)	0.0057 (9)
C18	0.0394 (13)	0.0816 (18)	0.0506 (14)	−0.0053 (12)	−0.0014 (11)	0.0011 (12)
C19	0.0539 (16)	0.104 (2)	0.0579 (16)	−0.0041 (15)	−0.0127 (13)	0.0004 (15)
N1	0.0328 (10)	0.0649 (13)	0.0754 (15)	0.0006 (9)	0.0003 (10)	0.0121 (11)
N2	0.0457 (11)	0.0590 (12)	0.0419 (11)	0.0019 (9)	−0.0016 (8)	−0.0020 (8)
N3	0.0417 (11)	0.0635 (12)	0.0427 (11)	0.0022 (9)	−0.0006 (8)	−0.0010 (9)
N4	0.0459 (13)	0.0927 (17)	0.0788 (17)	−0.0042 (11)	−0.0120 (11)	0.0011 (13)
O1	0.0483 (10)	0.0923 (14)	0.0722 (12)	−0.0078 (9)	−0.0216 (9)	−0.0066 (10)
O2	0.0431 (9)	0.0843 (13)	0.0613 (11)	−0.0048 (8)	0.0007 (8)	−0.0140 (9)
O3	0.0325 (8)	0.0515 (9)	0.0379 (8)	−0.0036 (6)	−0.0017 (6)	0.0002 (6)

### Geometric parameters (Å, °)

**Table:**

C1—O1	1.210 (2)	C11—C12	1.377 (3)
C1—O2	1.316 (3)	C11—H11	0.9300
C1—C2	1.493 (3)	C12—N1	1.323 (3)
C2—C7	1.387 (3)	C12—H12	0.9300
C2—C3	1.387 (3)	C13—N2	1.292 (3)
C3—C4	1.375 (3)	C13—O3	1.357 (2)
C3—H3	0.9300	C14—N3	1.287 (3)
C4—C5	1.372 (3)	C14—O3	1.360 (2)
C4—H4	0.9300	C14—C17	1.458 (3)
C5—C6	1.370 (3)	C15—N4	1.324 (3)
C5—H5	0.9300	C15—C16	1.380 (3)
C6—C7	1.384 (3)	C15—H15	0.9300
C6—H6	0.9300	C16—C17	1.381 (3)
C7—H7	0.9300	C16—H16	0.9300
C8—N1	1.332 (3)	C17—C18	1.369 (3)
C8—C9	1.369 (3)	C18—C19	1.374 (3)
C8—H8	0.9300	C18—H18	0.9300
C9—C10	1.378 (3)	C19—N4	1.331 (3)
C9—H9	0.9300	C19—H19	0.9300
C10—C11	1.385 (3)	N1—H1A	0.8600
C10—C13	1.459 (3)	N2—N3	1.407 (2)
			
O1—C1—O2	123.0 (2)	C10—C11—H11	120.7
O1—C1—C2	123.0 (2)	N1—C12—C11	122.4 (2)
O2—C1—C2	113.95 (18)	N1—C12—H12	118.8
C7—C2—C3	119.5 (2)	C11—C12—H12	118.8
C7—C2—C1	119.06 (19)	N2—C13—O3	112.41 (18)
C3—C2—C1	121.43 (19)	N2—C13—C10	128.76 (19)
C4—C3—C2	120.0 (2)	O3—C13—C10	118.84 (17)
C4—C3—H3	120.0	N3—C14—O3	112.67 (18)
C2—C3—H3	120.0	N3—C14—C17	129.31 (19)
C5—C4—C3	120.2 (2)	O3—C14—C17	117.96 (18)
C5—C4—H4	119.9	N4—C15—C16	124.6 (2)
C3—C4—H4	119.9	N4—C15—H15	117.7
C6—C5—C4	120.3 (2)	C16—C15—H15	117.7
C6—C5—H5	119.9	C15—C16—C17	118.4 (2)
C4—C5—H5	119.9	C15—C16—H16	120.8
C5—C6—C7	120.2 (2)	C17—C16—H16	120.8
C5—C6—H6	119.9	C18—C17—C16	118.0 (2)
C7—C6—H6	119.9	C18—C17—C14	121.20 (19)
C6—C7—C2	119.7 (2)	C16—C17—C14	120.8 (2)
C6—C7—H7	120.2	C17—C18—C19	119.1 (2)
C2—C7—H7	120.2	C17—C18—H18	120.5
N1—C8—C9	122.2 (2)	C19—C18—H18	120.5
N1—C8—H8	118.9	N4—C19—C18	124.4 (3)
C9—C8—H8	118.9	N4—C19—H19	117.8
C8—C9—C10	119.1 (2)	C18—C19—H19	117.8
C8—C9—H9	120.5	C12—N1—C8	119.1 (2)
C10—C9—H9	120.5	C12—N1—H1A	120.5
C9—C10—C11	118.7 (2)	C8—N1—H1A	120.5
C9—C10—C13	119.94 (19)	C13—N2—N3	106.18 (17)
C11—C10—C13	121.35 (18)	C14—N3—N2	106.04 (17)
C12—C11—C10	118.5 (2)	C15—N4—C19	115.6 (2)
C12—C11—H11	120.7	C13—O3—C14	102.71 (15)
			
O1—C1—C2—C7	−7.7 (3)	C15—C16—C17—C18	0.1 (4)
O2—C1—C2—C7	171.85 (19)	C15—C16—C17—C14	−178.0 (2)
O1—C1—C2—C3	172.7 (2)	N3—C14—C17—C18	−167.8 (2)
O2—C1—C2—C3	−7.7 (3)	O3—C14—C17—C18	9.0 (3)
C7—C2—C3—C4	0.0 (3)	N3—C14—C17—C16	10.2 (4)
C1—C2—C3—C4	179.54 (19)	O3—C14—C17—C16	−172.9 (2)
C2—C3—C4—C5	−0.5 (3)	C16—C17—C18—C19	−0.3 (4)
C3—C4—C5—C6	0.4 (4)	C14—C17—C18—C19	177.8 (2)
C4—C5—C6—C7	0.3 (4)	C17—C18—C19—N4	0.6 (4)
C5—C6—C7—C2	−0.8 (4)	C11—C12—N1—C8	−0.6 (3)
C3—C2—C7—C6	0.7 (3)	C9—C8—N1—C12	−0.9 (4)
C1—C2—C7—C6	−178.9 (2)	O3—C13—N2—N3	−0.5 (2)
N1—C8—C9—C10	1.5 (4)	C10—C13—N2—N3	179.14 (19)
C8—C9—C10—C11	−0.6 (3)	O3—C14—N3—N2	−0.4 (2)
C8—C9—C10—C13	179.7 (2)	C17—C14—N3—N2	176.6 (2)
C9—C10—C11—C12	−0.8 (3)	C13—N2—N3—C14	0.5 (2)
C13—C10—C11—C12	178.89 (19)	C16—C15—N4—C19	0.3 (4)
C10—C11—C12—N1	1.4 (3)	C18—C19—N4—C15	−0.5 (4)
C9—C10—C13—N2	2.8 (3)	N2—C13—O3—C14	0.2 (2)
C11—C10—C13—N2	−176.8 (2)	C10—C13—O3—C14	−179.42 (17)
C9—C10—C13—O3	−177.63 (18)	N3—C14—O3—C13	0.1 (2)
C11—C10—C13—O3	2.7 (3)	C17—C14—O3—C13	−177.24 (18)
N4—C15—C16—C17	−0.1 (4)		

### Hydrogen-bond geometry (Å, °)

**Table:**

*D*—H···*A*	*D*—H	H···*A*	*D*···*A*	*D*—H···*A*
N1—H1A···O2^i^	0.86	1.79	2.648 (2)	174
C8—H8···O1^ii^	0.93	2.48	3.371 (3)	161

Symmetry codes: (i) −*x*, *y*−1/2, −*z*+1/2; (ii) −*x*, −*y*+1, −*z*.
